# Polyphenol Extracts From Germinated Mung Beans Can Improve Type 2 Diabetes in Mice by Regulating Intestinal Microflora and Inhibiting Inflammation

**DOI:** 10.3389/fnut.2022.846409

**Published:** 2022-03-24

**Authors:** Xinting Shen, Xiujie Jiang, Lili Qian, Aiwu Zhang, Feng Zuo, Dongjie Zhang

**Affiliations:** ^1^National Coarse Cereals Engineering Research Center, Heilongjiang Bayi Agricultural University, Daqing, China; ^2^College of Food Science, Heilongjiang Bayi Agricultural University, Daqing, China

**Keywords:** T2DM, inflammation, intestinal flora, hypoglycemic, germinated mung bean polyphenols

## Abstract

Studies have shown that inhibiting inflammation and regulating intestinal microflora imbalance is a significant factor in controlling the development of type 2 diabetes mellitus (T2DM). This experiment studied the protective effect of polyphenol extract from germinated mung beans on diabetic C57BL/6 mice.

**Results:**

Fasting blood glucose (FBG) was decreased, glucose tolerance was increased, insulin resistance was decreased, serum lipid indexes in T2DM mice were improved, and the enzyme activities of alanine aminotransferase (ALT) and aspartate transaminase (AST) in serum were reduced. Meanwhile, the levels of interleukin 6 (IL-6), tumor necrosis factor-α (TNF-α), and C-reactive protein (CRP) in serum were decreased, the concentration of interleukin 10 (IL-10) in serum was increased, inhibiting the inflammatory reaction induced by diabetes and repairing the morphology of mice liver tissue. At the same time, germinated mung bean polyphenol (GMP) can regulate the main intestinal flora, Firmicutes, Bacteroidetes, and Proteobacteria in diabetic mice and can also regulate species diversity and improve intestinal flora imbalance. Taken together, the experimental conclusion is a certain dose of polyphenol extract from germinated mung beans that can improve mouse T2DM by inhibiting inflammatory reaction and regulating intestinal microflora.

## Introduction

Type 2 diabetes mellitus (T2DM) is a chronic disease accompanied by an inflammatory response, commonly referred to as non-insulin-dependent diabetes mellitus (1). The main features are increased blood glucose and insulin resistance (2, 3). According to WHO data, T2DM accounts for 90–95% of the cases, and its rapid growth has always been an international issue (4). T2DM has different degrees of the chronic low-level inflammatory response (5). The release of inflammatory factors may lead to islets β-cell structure and function is damaged, and even insulin secretion and transport function are impaired (6). Most treatments for T2DM are to stimulate insulin secretion, reduce glucose production, and enhance the effect of insulin on target tissues to reduce blood glucose levels (7, 8). In recent years, many studies have found that intestinal flora is also a critical factor involved in the treatment of diabetes, and it is found that it is connected to inflammation, insulin resistance, and islet function. Because of its sensitive reaction to factors, it is considered to be an important valuable tool for developing and promoting human health.

Legumes are considered to be the second most important food crops. Mung bean is one of the beans that people often eat in China (9) and is recommended for diabetics because of its high fiber content and low sugar content. At present, there is evidence that mung beans can ameliorate blood glucose, blood lipid, and blood pressure, protect the liver, and regulate immune activity. The beneficial effects mainly come from the active compounds in mung beans. It is reported that the active components of mung beans, especially polyphenols, have health benefits (10–12). As one of the food-processing methods, germination can significantly improve the nutritional value and the content of bioactive components of mung beans. Mung bean has more functional substances after germination (13), and its internal macromolecular substances will degrade into small molecules, which is more conducive to human absorption. The process of Mung Bean Germination is essentially the activation process of mung beans. Phenols are the products of secondary metabolism. They have health benefits (14), can reduce some risk factors of T2DM, and also show some anti-inflammatory effects (15, 16). *In vitro* and clinical studies have shown that polyphenols can regulate intestinal microbiota, conducive to intestinal health changes, reduce inflammatory process, and improve physical health indicators. It is considered as a new treatment strategy to treat various metabolic diseases, such as T2DM, by regulating intestinal flora and improving inflammatory response (17).

Therefore, mung bean was used as raw material in this experiment. After germination, polyphenol extract was obtained after extraction and purification. A high-fat diet (HFD) combined with an intraperitoneal injection of Streptozotocin (STZ) was used to establish a mouse model of T2DM. T2DM mice were fed with different concentrations of germinated mung bean polyphenols (GMPs) extract. The levels of fasting blood glucose (FBG), glucose tolerance, insulin level, blood lipid level, inflammatory factors, and liver tissue sections were analyzed. The aim of the study was to explore the effect of GMP extract on improving glucose and lipid metabolism and inhibiting inflammation in T2DM mice. The intestinal flora of mice was analyzed by Illumina NovaSeq and explored the relationship between diabetes and intestinal flora and inflammatory factors. This experiment provides experimental evidence for improving T2DM and also meets the demand for natural, safe, and effective hypoglycemic functional foods in the market at present.

## Materials and Methods

### Materials and Reagents

Mung bean was purchased from Chifeng, Inner Mongolia, GMP extract is made by the laboratory of Food College of Heilongjiang Bayi Agricultural Reclamation University; STZ was purchased from Sigma of the United States; metformin hydrochloride was purchased from Shanghai Yuanye Biotechnology Co., Ltd. (>99.9% purity), and basic feed and high-fat feed were purchased from TROPHIC Animal Feed High-Tech Co. LTD, China. Feed code: TP23300.

### Preparation of Polyphenol Extract From Germinated Mung Bean

Mung bean with good fresh quality was selected for germination. Using the response surface optimization method, the optimum germination process of mung bean was determined as follows: soaking temperature 25°C, soaking time 11 h, germination temperature 28°C, germination time 3 d, and CaCl_2_ concentration of soaking solution 2 mmol/L. Under these conditions, the polyphenol content was 487.81 mg/100 g. It is three times the content of mung beans. Mung beans are germinated, taken out and washed drying (40°C, blast drying oven), and crushing (80 mesh screening). Weigh the screened germinated mung bean powder into a beaker, add 70% ethanol solution according to the material-liquid ratio parameter of 1:26 (g/ml), take the supernatant through ultrasound, centrifugation, rotate, and evaporate to dryness at 45°C to obtain the crude polyphenol extract of germinated mung bean (18–21). Purify the crude polyphenol extract (AB-8 macroporous resin), collect the purified solution, and freeze-drying to obtain the GMP.

### Animals and Experimental Design

These experiments were carried out on 36 male C57BL/6 mice (4 weeks old), with an average weight of (18–20) g, provided by Liaoning Changsheng Biotechnology Co., Ltd. (Liaoning, China). The animal use license number is scxk (Liaoning) 2020-0001. The mice were placed at room temperature (23 ± 2°C), relative humidity (45 ± 5%), and light (12 h/12 h light/dark), cage feeding was carried out in a well-ventilated environment, and free eat food and drink water was adopted throughout the whole experiment.

A high-fat diet combined with an intraperitoneal injection of STZ was used in the experiment (30 mg/kg BW) to establish type 2 diabetic mice. Specific pathogen free (SPF) grade male mice were fed with the basal diet for 1 week. According to body weight, 30 mice were randomly selected as a diabetic model group. After 4 weeks, all mice were fasted 12 h later and injected with STZ. Normal mice were intraperitoneally injected with the same volume of citric acid buffer (pH = 4), continuously injected two times, and FBG was measured after 3 days. Blood was collected by caudal vein. After 1 week, the FBG in mice was more than 11.1 mmol/L for 7 days, and the type 2 diabetes mouse model was successfully established.

Six mice were randomly selected and fed normally as the control group (N). Diabetic mice were randomly divided into T2DM model group (M), low dose intervention group (GMPL), medium dose intervention group (GMPM), and high dose intervention group (GMPH). The low (50 mg/kg), medium (100 mg/kg), and high (150 mg/kg) dose intervention groups were given GMP every day. N group and M group received saline by gastric perfusion, for 5 weeks. Throughout the experiment, the diet, mental state, hair color, urine volume, body weight, and blood glucose of mice were observed. After intragastric administration for 5 weeks, fresh feces of mice in each group were collected in sealed sterile plastic tubes. Feces are frozen at −80°C for standby. After the last intervention, the mice fasted for 6 h, intragastric administration of 2 g/kg glucose (concentration 25%), blood glucose levels were measured at 0, 30, 60, 90, and 120 min after gavage. Before the end of the experiment, all mice fasted for 12 h, measured FBG and body weight, and then were anesthetized with ether. The eyeballs were taken out for blood. The blood was centrifuged at 3,500 r/min for 10 min, and the separated serum was stored at −80°C for index detection. Under anesthesia, the mice were dissected, taken out, and weighed to measure liver tissue. The left liver was fixed with 4% paraformaldehyde for histopathological analysis. The right liver was immediately frozen at −80°C for the determination of other indexes.

### Serum Biochemical Analysis

The concentrations of total cholesterol (TC), triglycerides (TG), high-density lipoprotein (HDL-C), high-density lipoprotein (LDL-C), alanine aminotransferase (ALT), and aspartate transaminase (AST) in serum were measured by kit, and the levels of inflammatory factors interleukin (IL)-6, IL-10, tumor necrosis factor-α (TNF-α), and C-reactive protein (CRP) were measured by ELISA kit. Start the operation according to the instructions of the kit. The kit was purchased from Nanjing Jiancheng Biotechnology Company (Nanjing, China). Homeostasis model assessment insulin resistance index (HOMA-IR) is often used to observe the level of insulin resistance. Calculation formula: HOMA-IR = FBG (mmol/L) × fasting insulin (FINS) (μU/ml)/22.5 (22). Homeostasis model assessment-β calculation formula: HOMA-β = (20 × FINS)/(FBG–3.5) (23).

### Histological Analysis of Liver

After dissection, some liver tissues were fixed with 4% paraformaldehyde solution. The fixed liver tissues were embedded in paraffin, sectioned, and stained with hematoxylin-eosin (H&E). The liver injury and inflammatory cell infiltration were observed under a light microscope.

### Analysis of Intestinal Flora

The feces of 4 mice in each group were randomly selected for intestinal flora analysis. Mouse fecal samples were tested by Beijing Baimaike Co., Ltd. for amplification, purify, quantify, and homogenize the products, form a sequencing database, and sequence them with Illumina NovaSeq 6,000.

### Statistical Analysis

The data were expressed as mean ± SD by origin (2018) software and statistically analyzed by SPSS (26.0). *p* < 0.05 was statistically significant.

## Results

### Effects of GMP on Body Weight and Liver Index in T2DM Mice

**Figure 1:**
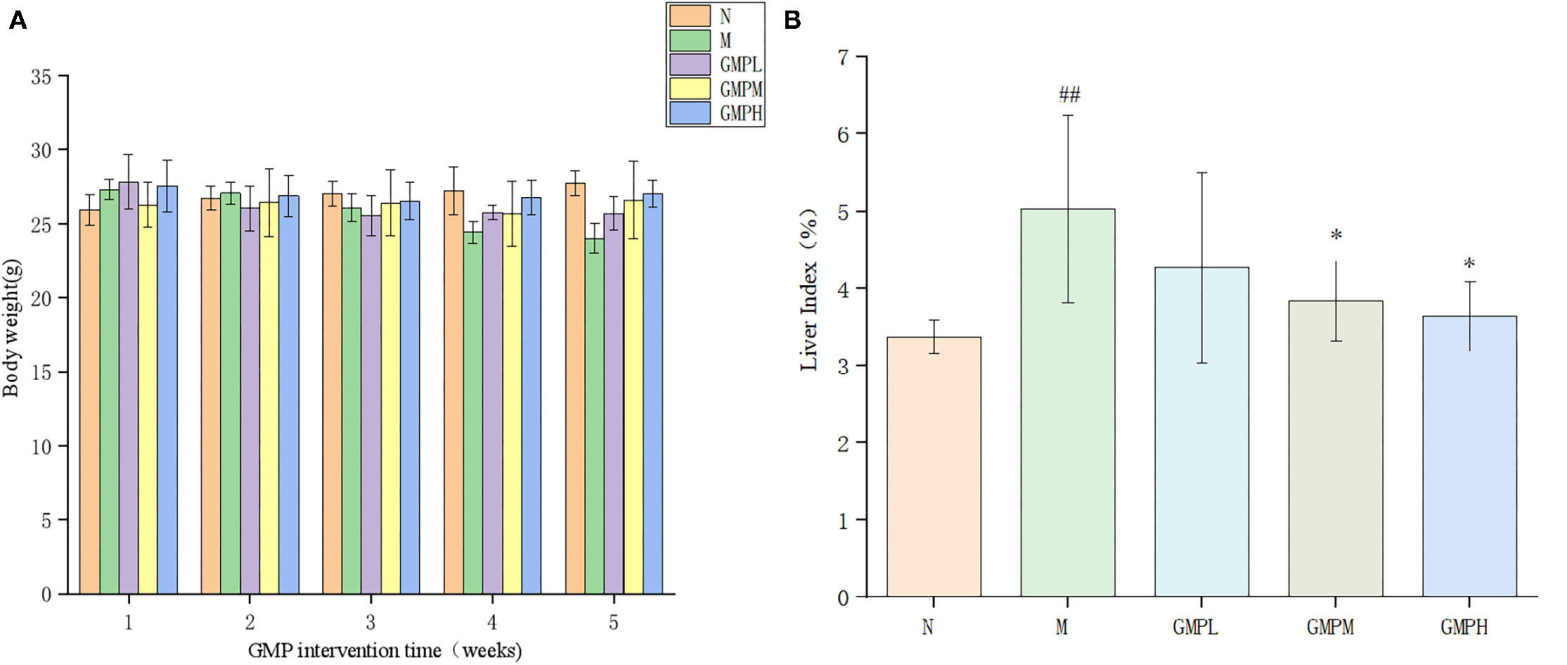
Changes of body weight and liver index during GMP intervention. **(A)** Body weight. **(B)** Liver index. The data in the figure are expressed as mean ± SD (*n* = 6). Significance of difference: *means *p* < 0.05, **means *p* < 0.001, compared with group M. ^##^means *p* < 0.001, compared with group N. GMP, germinated mung bean polyphenol.

The body weight and liver index of mice in the normal group and experimental group are shown in Figure 1. In the last week of GMP intervention, compared with group N, the weight of mice in the T2DM model group was declined significantly (Figure 1A). The weight of GMPM and GMPH intervention groups was increased significantly compared with group M.

The liver index shows the relationship between liver weight and body weight of experimental animals and reflects the pathological phenomenon of swelling or atrophy of liver tissue in mice. Compared with group N, the liver index of mice in group M was increased significantly by 49.26%; compared with group M, the liver index of the GMPM group and GMPH group was decreased significantly (Figure 1B). The results show that T2DM mice will have obvious liver swelling, and GMP intervention can reduce the liver swelling of T2DM mice.

### Effect of GMP on Glucose and Lipid Metabolism in T2DM Mice

**Figure 2:**
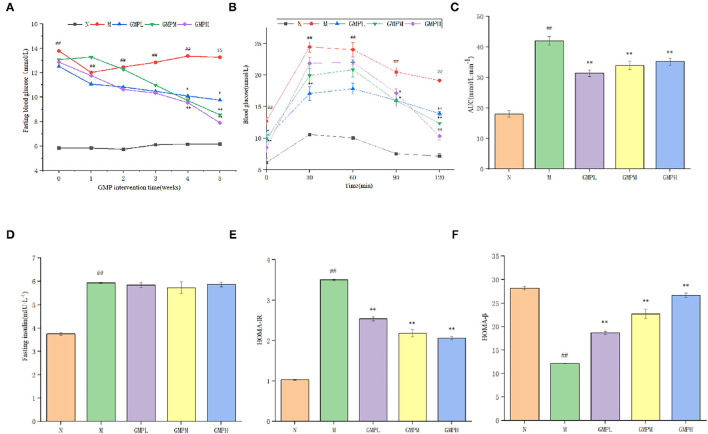
Effects of different doses of GMP on **(A)** fasting blood glucose levels. **(B)** Oral glucose tolerance. **(C)** Blood glucose area under curve. **(D)** Fasting serum insulin levels. **(E)** HOMA-IR. **(F)** HOMA-β. The data in the figure are expressed as mean ± SD (n = 6). Significance of difference: *means *p* < 0.05, **means *p* < 0.001, compared with group M. ^##^means *p* < 0.001, compared with group N. GMP, germinated mung bean polyphenol; HOMA-IR, Homeostasis model assessment insulin resistance index.

The effect of GMP on blood glucose in diabetic mice is shown in Figure 2A. The blood glucose level in group N was stable. Blood glucose level of group M was increased gradually after the establishment of the diabetes model. Compared with group M, FBG in GMPM and GMPH groups was decreased significantly after 4 weeks of GMP intervention. At the end of the whole experiment, the FBG concentration in GMPL, GMPM, and GMPH groups was significantly less than that in group M. The measurement results of FBG showed that GMP could improve the FBG level of T2DM mice during the intervention. In addition, the intervention effect of the GMPH group was better than that of other intervention groups.

Glucose tolerance refers to the body's ability to tolerate glucose according to the degree of glucose tolerance, judge the ability of islet β cells to regulate blood glucose (24). Blood glucose area under curve (AUC) can indicate the degree of glucose utilization and clearance by the human body. If the number increases, it indicates that the glucose tolerance of the body decreases, on the contrary, it indicates that the glucose tolerance of the body increases. The FBG concentration of mice in group N tended to change gently with time (Figure 2B). The blood glucose in group M was increased rapidly after 30 min of intragastric glucose, reached the peak, and then was decreased, but the FBG level was relatively high. The blood glucose level of each intervention group was decreased slowly after 60 min, but did not return to the initial blood glucose. AUC in group M was higher than that in group N; compared with group M, the performance of each intervention group was significantly lower (Figure 2C). The results showed that GMP could improve glucose tolerance of T2DM mice to a certain extent, and the recovery effect of the GMPH group was better in terms of recovery degree.

Insulin is the only hormone secreted by islet β cells that can reduce blood glucose and has an important effect on regulating blood glucose. As shown in Figures 2D,E, compared with group N, FINS level and HOMA-IR index in group M have increased significantly; compared with group M, different doses of GMP intervention group significantly reduced HOMA-IR index level. Although the insulin level decreased, there was no significant difference. HOMA-β is an important indicator (25) that can evaluate islets β cell function. When diabetes becomes serious, it decreased islet β cell function (26). As shown in Figure 2F, compared with group N, the HOMA-β index of group M has decreased significantly. Compared with group M, the HOMA-β index of each dose of the GMP intervention group can be significantly improved. According to the results of Figure 2, GMP can increase insulin sensitivity, reduce insulin resistance, and repair damaged islets β cells function in T2DM mice.

**Figure 3:**
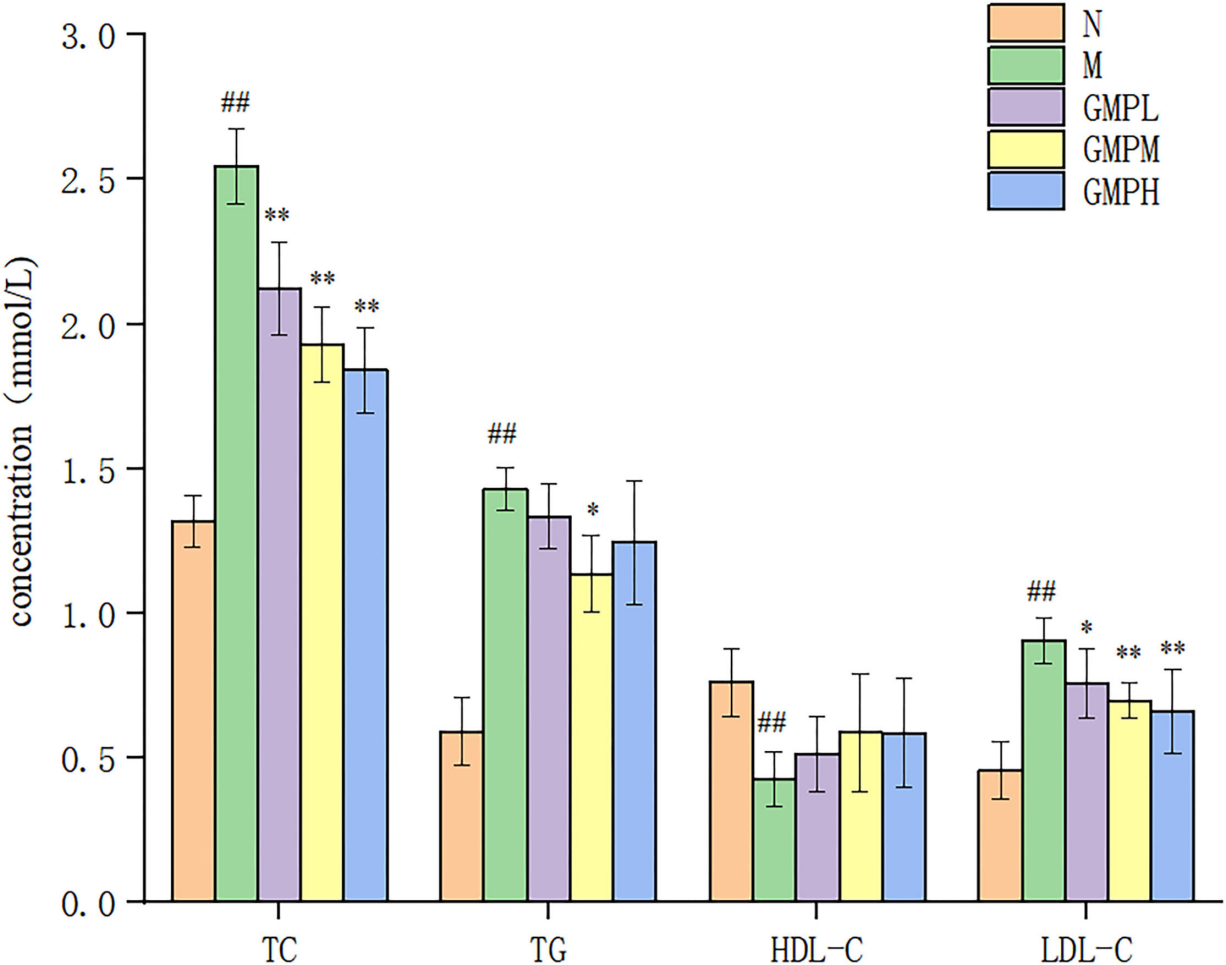
Effects of different doses of GMP on TC, TG, HDL-C, and LDL-C levels. The data in the figure are expressed as mean ± SD (*n* = 6). Significance of difference: *means *p* < 0.05, **means *p* < 0.001, compared with group M. ^##^means *p* < 0.001, compared with group N. GMP, germinated mung bean polyphenol; TC, total cholesterol; TG, triglycerides; HDL-C, high-density lipoprotein; HDL-C, low-density lipoprotein (LDL-C), ALT, alanine aminotransferase; AST, aspartate transaminase.

Diabetes is associated with blood lipid levels, and the lowering of blood lipids helps to improve diabetes. Compared with group N, the concentrations of TC, TG, and LDL-C in group M mice were significantly increased, and the concentration of HDL-C was decreased (Figure 3). The results showed that T2DM mice had lipid metabolism disorder. Compared with group M, the TC concentration in GMPL, GMPM, and GMPH groups was decreased by 16.86, 24.31, and 27.84%, respectively (*p* < 0.01), the LDL-C concentration was also decreased significantly, and the TG concentration in the GMPM group was also decreased by 20.49%. The results showed that GMP could regulate the disorder of lipid metabolism in T2DM mice.

### Effect of GMP on Serum Indexes in T2DM Mice

**Figure 4:**
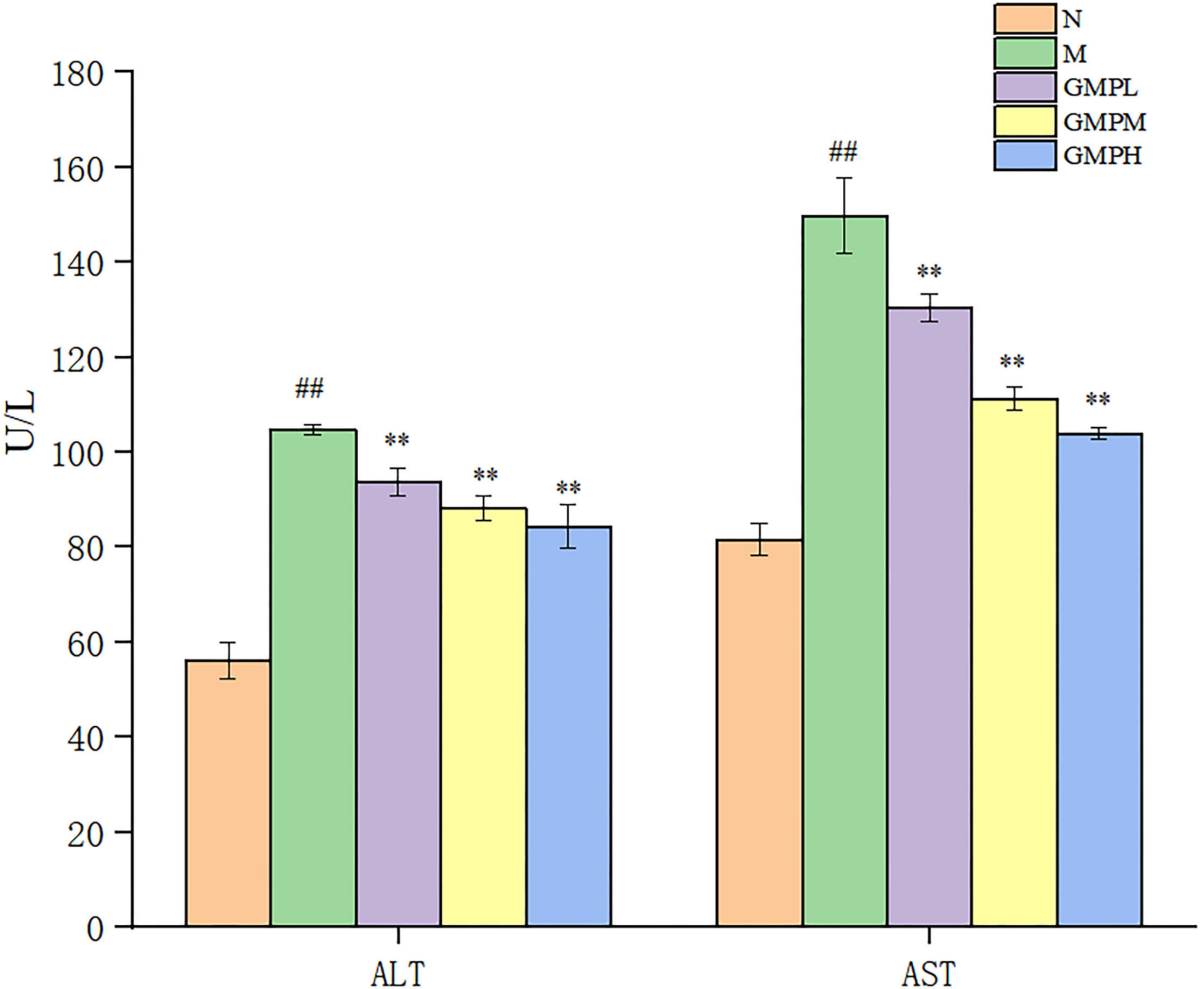
Effects of different doses of GMP on ALT and AST. The data in the figure are expressed as mean ± SD (*n* = 6). Significance of difference: *means *p* < 0.05, **means *p* < 0.001, compared with group M. ^##^means *p* < 0.001, compared with group N. GMP, germinated mung bean polyphenol; ALT, alanine aminotransferase; AST, aspartate transaminase.

Alanine aminotransferase and AST enzyme activities are necessary indexes to reflect liver function. However, liver pathology can more intuitively judge the degree of tissue damage (27). Compared with group N, the ALT and AST activities in mice M were increased, and compared with group M, the ALT and AST activities in the GMP intervention group were decreased significantly (Figure 4). The results showed that GMP could reduce the activity of serum ALT and AST in mice.

**Figure 5:**
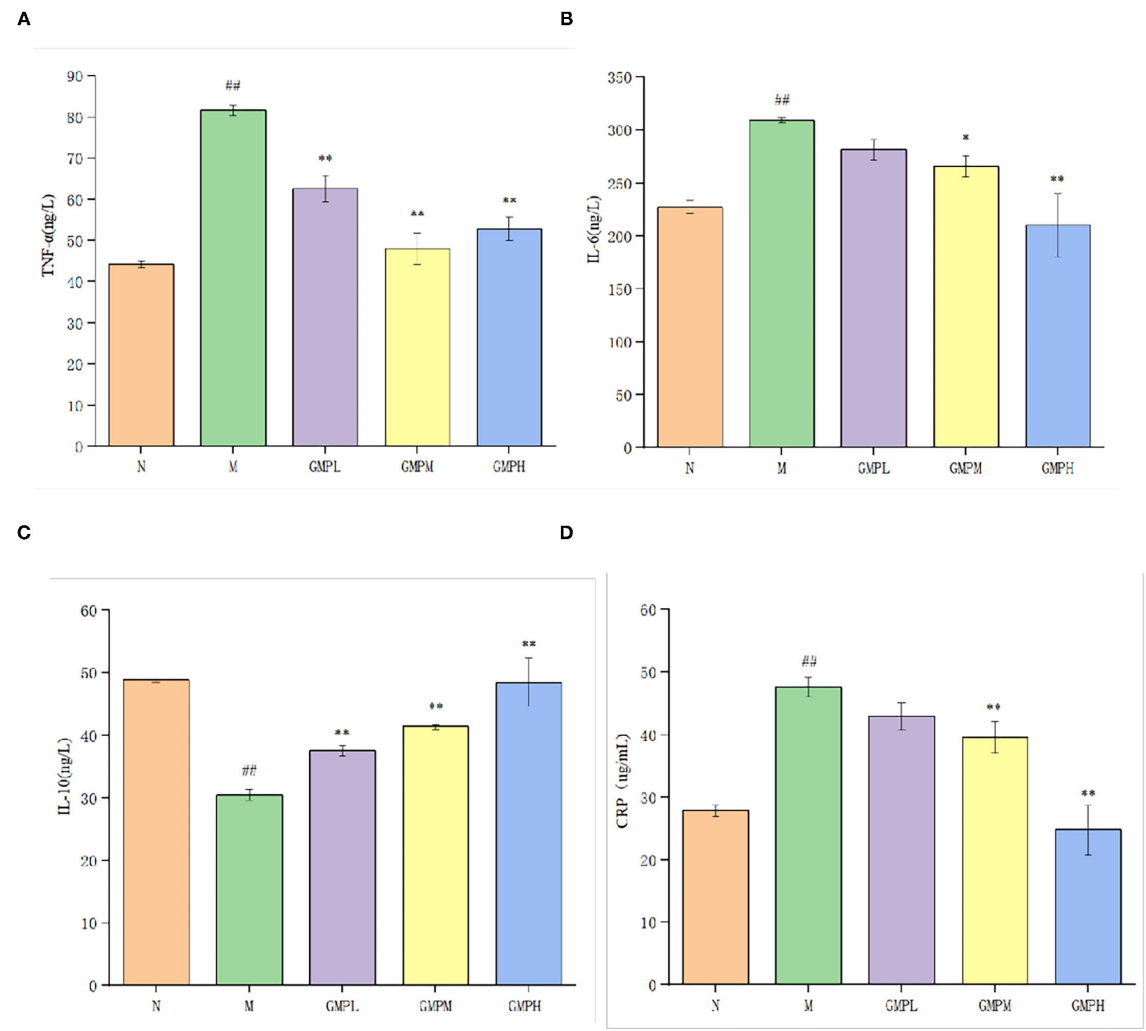
Effects of different doses of GMP on serum inflammatory factors in mice. **(A)** Serum TNF-α level. **(B)** Serum IL-6 level. **(C)** Serum IL-10 level. **(D)** Serum CRP level. The data in the figure are expressed as mean ± SD (*n* = 6). Significance of difference: *means *p* < 0.05, **means *p* < 0.001, compared with group M. ^##^means *p* < 0.001, compared with group N. GMP, germinated mung bean polyphenol; IL, interleukin, TNF-α, tumor necrosis factor-α.

The effect of GMP on the inflammatory response of T2DM mice is shown in Figure 5. Compared with group N, IL-6 in serum of group M mice was increased from 227.37 to 309.25 ng/L, TNF-α from 44.22 to 81.64 ng/L, CRP was increased from 27.84 to 47.68 μg/ml, and IL-10 was decreased significantly. CRP is a non-specific inflammatory marker. When the CRP is 10–50 mg/L, it indicates mild inflammation. Compared with group N, group M had an inflammatory reaction.

Compared with group M, the concentrations of IL-6 and CRP in GMPM and GMPH groups were decreased significantly. Although they decreased in GMPL, the difference was not significant. The concentration of TNF-α was decreased significantly and the concentration of IL-10 was increased significantly in low, medium, and high dose intervention groups. The results showed that the GMP intervention group could further reduce the inflammatory reaction induced by diabetes by reducing the levels of IL-6, TNF-α, and CRP and increasing IL-10.

### Effect of GMP on Liver Pathology in T2DM Mice

**Figure 6:**
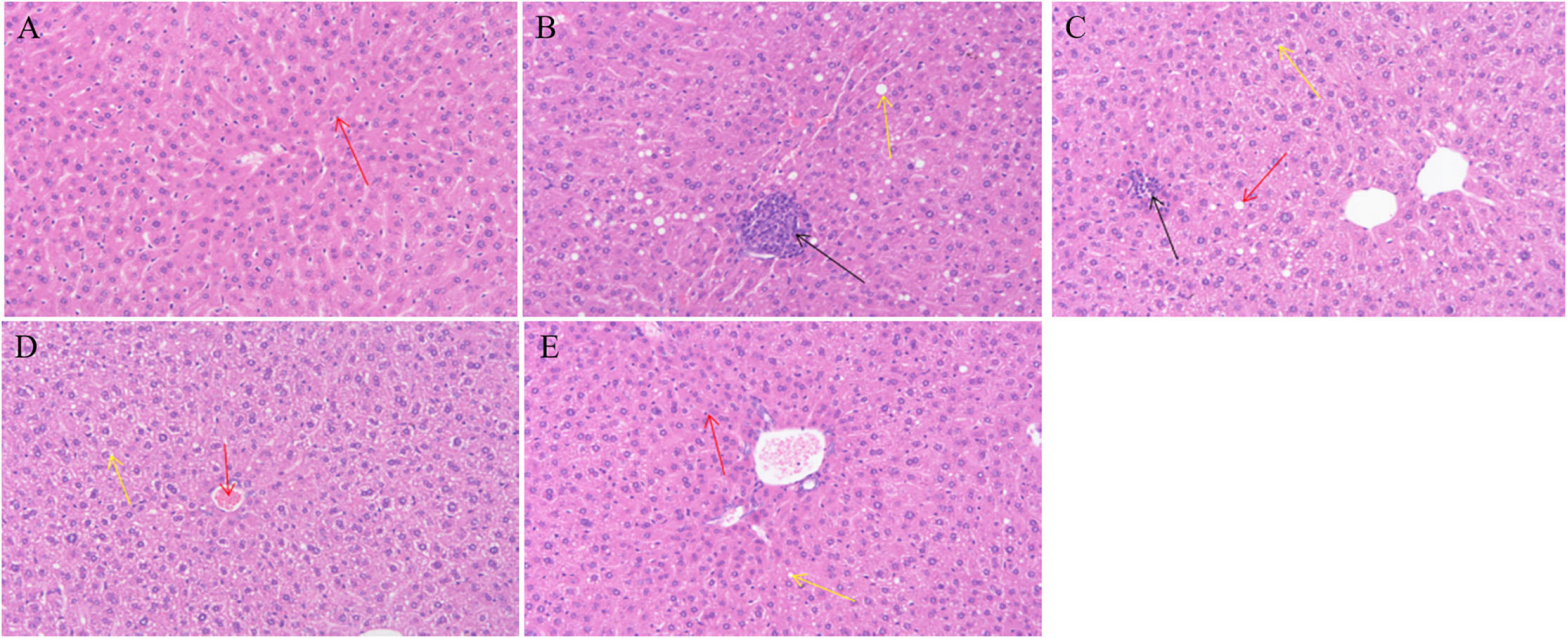
Effects of different doses of GMP on liver tissue of mice. **(A)** Normal group. **(B)** T2DM model group. **(C)** GMPL group. **(D)** GMPM group. **(E)** GMPH group. Magnification: **(A–E)** ×100. GMP, germinated mung bean polyphenol; T2DM, type 2 diabetes mellitus; GMPL, low dose intervention group; GMPM, medium dose intervention group; GMPH, high dose intervention group.

Compared with group M, there was no serious injury to the liver tissue of animals, indicating that the gavage dose of GMP is safe for animals. As shown in Figure 3, the liver tissue structure of group N is basically normal, no inflammatory cell infiltration is found, the liver cell structure is complete, the central vein of the tissue is clearly visible, the hepatic sinus is radial along the central vein, and the red arrow shows the macrophages of the hepatic sinus. Group M had abnormal liver tissue structure, loose hepatocyte structure, and more inflammatory cell infiltration (as shown by the black arrow in Figure 6). In the GMPL group, some hepatocytes are slightly edematous, and obvious inflammatory cell infiltration is seen in the tissue, as shown by the black arrow in Figure 6. GMPM liver tissue structure is slightly abnormal, no obvious inflammatory cell infiltration is found, and some hepatocytes are slightly edematous, as shown by the yellow arrow in Figure 6. In the GMPH group, the liver tissue structure was basically normal, the hepatocyte structure was full, the central vein of the tissue was clearly visible, and there was no obvious inflammatory cell infiltration.

### Effect of GMP on Intestinal Flora in Diabetic Mice

#### Analysis of Alpha Diversity in the Mouse Intestine

**Figure 7:**
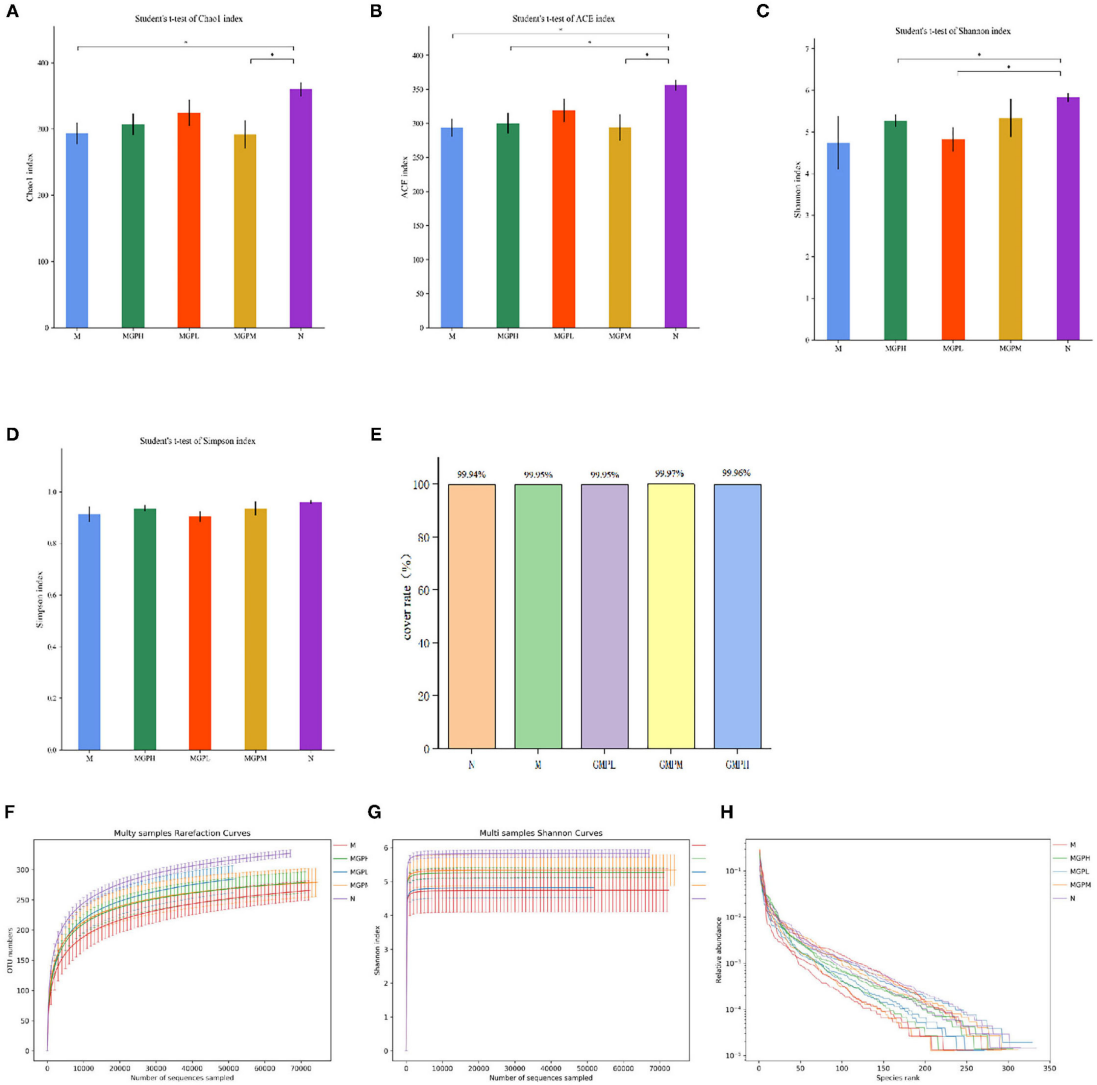
Changes of intestinal flora in mice α diversity index. **(A)** Chao index. **(B)** Ace index. **(C)** Shannon index. **(D)** Simpson index. **(E)** Cover rate. **(F)** Rarefaction Curve. **(G)** Shannon index. **(H)** Rank Abundance Curve. Significance of difference: *means *p* < 0.05, **means *p* < 0.001, compared with group M. ^##^means *p* < 0.001, compared with group N.

Alpha diversity shows species abundance and diversity of samples. As shown in Figure 7, the indexes of ACE, Chao, Shannon, and Simpson of intestinal flora in the M group are lower than in the N group, indicating that the species diversity of T2DM mice is reduced. Compared with group M, GMP could improve the intestinal flora α diversity index that can inhibit the decline of intestinal flora richness and increase the species diversity of flora. This experiment also counts the coverage rate, indicating that the sequencing results of the experiment represent the real situation of microorganisms in the sample. The higher the coverage rate, the greater the probability of species being detected. It shows that GMP intervention can balance the imbalance of intestinal flora to a certain extent.

A rarefaction curve can be used to judge whether the sample sequencing quantity is sufficient. As shown in Figure 7F, within the scope of the experiment, with the increase of sequencing times, the curve rises rapidly, indicating that many new species have been found; if the curve is flat, it indicates that the species in the environment will not increase with the increase of sequencing times. Shannon index curve shows the microbial diversity in samples with different sequencing quantities. The greater the Shannon index, the richer the species. As shown in Figure 7G, when the curve is flat, it indicates that the amount of data sequenced are sufficient, and the species will not increase. Increased sequencing times, Rank Abundance Curve can describe the abundance and consistency of species in the sample. As shown in Figure 7H, the wider the curve, the more the species composition, and the flat curve indicates that the uniformity of species composition is higher.

#### Taxonomic Analysis of Intestinal Microbial Species in Mice

**Figure 8:**
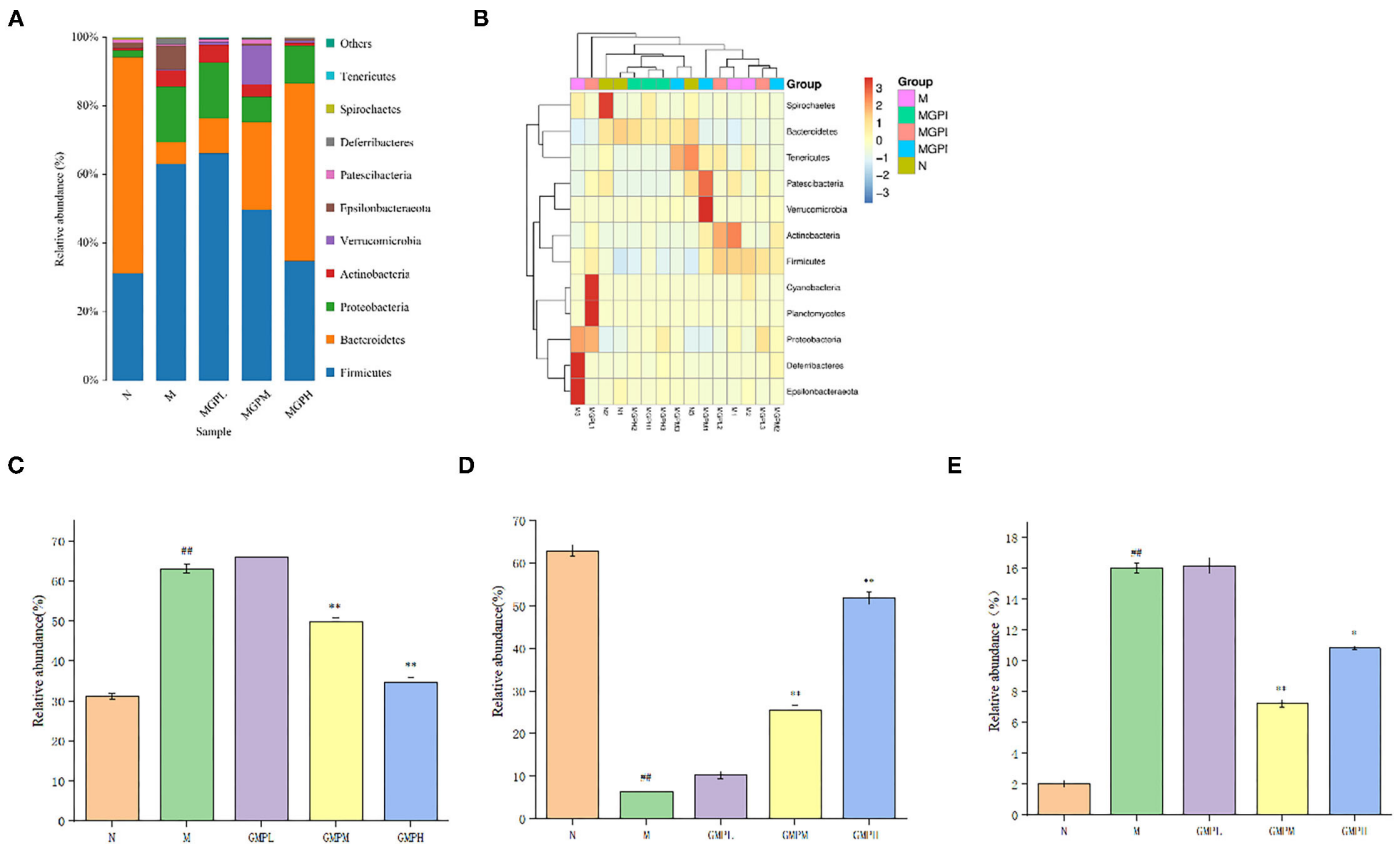
Analysis of phylum level of intestinal flora in mice. **(A)** Phylum level species distribution. **(B)** Heat map of species distribution at the gate level. **(C)** Firmicutes. **(D)** Bacteroidetes. **(E)** Proteobacteria. Significance of difference: *means *p* < 0.05, **means *p* < 0.001, compared with group M. ^##^means *p* < 0.001, compared with group N.

As shown in Figure 8, the comparative analysis of each group at the gate level shows that the intestinal flora mainly includes Firmicutes, Bacteroidetes, Proteobacteria, Actinobacteria, Verrucomicrobia, etc. The number of Firmicutes and Bacteroides in group M was significantly higher than that N group (*p* < 0.01). After GMP intervention, the number of Firmicutes were decreased significantly compared with group M, the abundance of Bacteroidetes was increased significantly, and the abundance of Proteus was decreased significantly. In short, the intestinal flora can balance in diabetic mice, and GMP intervention can regulate the abundance and diversity of T2DM mice.

### Correlation Analysis of Glucose and Lipid Metabolism Indexes, Inflammation Indexes, and Intestinal Flora

**Figure 9:**
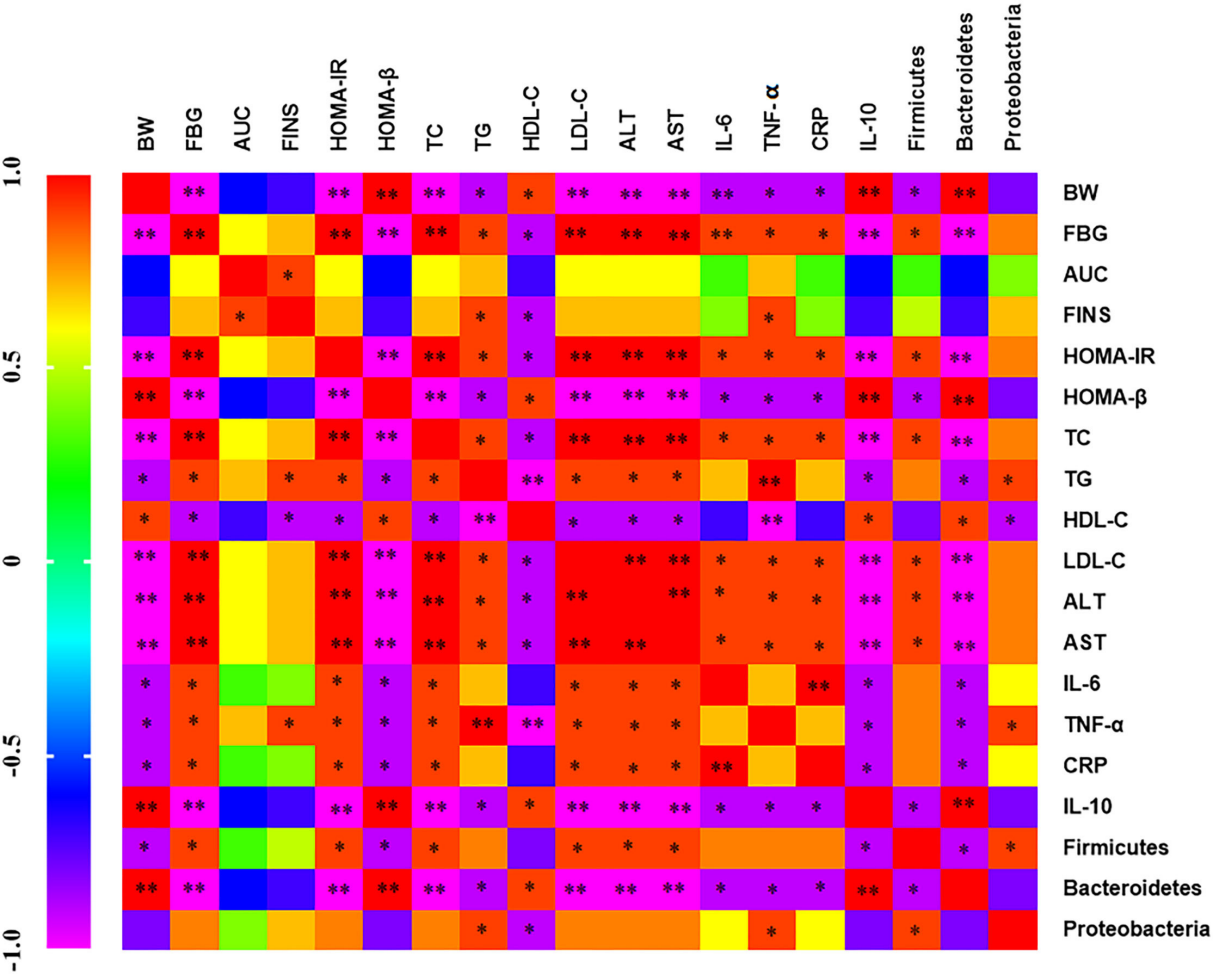
Heat map of correlation analysis between serum inflammatory factors, intestinal flora, and metabolic indexes Purple indicates negative correlation and red indicates positive correlation.*p < 0.05 and **p < 0.001, respectively.

The correlation between serum inflammatory factors, intestinal flora, and metabolic indexes was analyzed by Spearman. There is a positive correlation between blood glucose index (FBG, AUC, FINS, and HOMA-IR) and blood lipid index (TC, TG, and LDL-C), indicating that blood glucose index increases and blood lipid level also increases (Figure 9). IL-10 and Bacteroidetes were negatively correlated with blood glucose and lipid index, ALT, AST, IL-6 and TNF-α, CRP, Firmicutes, and Proteobacteria were positively correlated with blood glucose and lipid index. Firmicutes, Proteobacteria and IL-6, TNF-α, CRP were positively correlated and negatively correlated with IL-10. Bacteroidetes and IL-6, TNF-α, CRP were negatively correlated and positively correlated with IL-10. It indicates that inflammatory factors and intestinal flora are closely related to diabetes mellitus.

## Discussion

This experiment discusses the molecular mechanism of GMP improving T2DM from the perspective of intestinal microbiota and inflammatory factors. The results showed that GMP could improve glucose and lipid metabolism in T2DM mice induced by HFD and STZ, reduce hyperglycemia and insulin resistance, enhance glucose tolerance, repair liver tissue damage, and inhibit the inflammatory response. Further studies show that the mechanism of GMP regulating blood glucose may be related to the regulation of intestinal microbiota. 16SrRNA high-throughput sequencing showed that GMP could significantly decrease the abundance of Firmicutes and Proteobacteria and promote the proliferation of Bacteroidetes.

Type 2 diabetes mellitus is a metabolic disease based on the diagnosis of continuous hyperglycemia (28). The most obvious feature is glucose and lipid metabolism disorder, which can lead to pathological changes in tissues, organs, and digestive system (29), which is mainly caused by genetic and environmental factors, and the morbidity rate has continued to rise in recent years. Although the commonly used drugs for diabetes treatment in the market have good effects, most of them have some side effects. Therefore, the study of a natural and harmless good hypoglycemic functional food will soon become the focus of the current study of diabetes demand. Studies have shown that polyphenols extracted from beans (30), corn (31), black rice (32), and black tea (33) can inhibit glucose transport. Intake of polyphenol-rich diet can significantly reduce the risk of diabetes (34), which is due to polyphenols beneficial to glucose metabolism, can significantly improve glucose tolerance (35). This experiment found that GMPs can also reduce blood sugar, improve insulin resistance, and enhance glucose tolerance, which is consistent with the research conclusions mentioned above. Diabetes is closely related to blood lipid levels. Lowering blood lipid levels will help improve diabetes. It has been reported that HDL-C decreases and TC, TG, and LDL-C increase has adverse effects on mice (36). The results showed that the germination of mung bean polyphenol extract could reduce the levels of TC, TG, and LDL-C in serum of diabetic mice, increase HDL-C level, have a good regulatory effect on lipid metabolism disorder in T2DM mice, and it has positive effects on regulating the development of diabetes.

Diabetic patients will produce chronic inflammation in the body. TNF-α, IL-6, and IL-10 are inflammatory factors secreted by inflammatory cells (37). TNF-α, as the earliest inflammatory factor in the process of body inflammatory, plays an important role in glycoprotein signal transduction and synthesis. The level of TNF-α in patients with abnormal glucose tolerance increases significantly; IL-6 can control the signal transduction of insulin receptor and reduce insulin sensitivity. CRP is a non-specific inflammatory marker, which is synthesized by hepatocytes. The most important regulatory factors of CRP synthesis are IL-6 and TNF-α. The degree of its increase reflects the size and activity of inflammatory tissue. The liver is the key organ of metabolism and participates in the storage and emission of metabolites (38). Experiments show that GMP can reduce the levels of IL-6, TNF- α, and CRP, increase the level of IL-10, and then alleviate the inflammatory reaction induced by diabetes and repair the phenomenon of hepatocyte lysis and inflammatory cell infiltration in mice, so as to improve the liver injury. Studies have proved that germinated mung bean is an effective liver protective agent, which can reduce liver enzyme activity and repair damaged liver tissue within the experimental dose range (39).

In recent years, many animal and clinical experiments have shown that polyphenols can regulate the intestinal flora and the proportion of Firmicutes to Bacteroides (F/B) (40–42). The first organ contacted after dietary intake is the gastrointestinal tract, so the intestinal tract may be the main place where polyphenols play a metabolic role (43). The improvement of blood glucose and the regulation of blood lipid in this study may be closely related to the inhibition of the level of inflammatory factors in serum and the regulation of intestinal flora richness. One of the reasons why GMP can have an anti-inflammatory effect may be the regulation of intestinal flora. It was found that the higher the abundance of Firmicutes and Proteobacteria, the higher the levels of blood glucose and inflammatory indexes. The abundance of Bacteroidetes increased and the levels of blood glucose and inflammatory indexes decreased. It is reported that Bacteroidetes have anti-inflammatory and immune regulating effects, and the number of Bacteroidetes is reduced in the intestinal flora of the inflammatory mouse model (44, 45). The species diversity and abundance of intestinal flora in T2DM mice are closely related to the levels of blood glucose and inflammatory factors. Improving T2DM metabolic diseases by regulating intestinal flora and improving inflammatory response is considered to be a new treatment strategy (17).

## Conclusion

In summary, the results show that GMP can increase the intestinal microbial diversity, balance the dysfunctional intestinal flora, reduce inflammation and repair the liver injury, regulate the disorder of glucose and lipid metabolism, and finally reduce the blood glucose and blood lipid levels of T2DM mice. The results showed that the polyphenol extract from germinated mung bean is a natural hypoglycemic substance with potential and research value and has potential application value in the treatment of diabetes.

## Data Availability Statement

The original contributions presented in the study are publicly available. This data can be found here: https://www.ncbi.nlm.nih.gov/search/all/?term=PRJNA796743.

## Ethics Statement

The animal study was reviewed and approved by the Committee on Animal Care of the College of Animal Science and Veterinary Medicine of Heilongjiang Bayi Agricultural University.

## Author Contributions

DZ and AZ provided experimental plans and ideas. XS conducted experiments, sorted out data, and drew drawings. XJ, LQ, and FZ helped analyze data and participated in paper writing. All authors have read and agreed to submit the manuscript.

## Funding

Natural Science Fund Research Team Project of Heilongjiang Provincial Department of Science and Technology (TD2020C003); National Key R&D Plan, National Key R&D Plan of the Ministry of Science and Technology (2018YFE0206300).

## Conflict of Interest

The authors declare that the research was conducted in the absence of any commercial or financial relationships that could be construed as a potential conflict of interest.

## Publisher's Note

All claims expressed in this article are solely those of the authors and do not necessarily represent those of their affiliated organizations, or those of the publisher, the editors and the reviewers. Any product that may be evaluated in this article, or claim that may be made by its manufacturer, is not guaranteed or endorsed by the publisher.
